# Phylogenetic Analysis and Public Health Implications of *Salmonella* Strains in Southwestern States of Nigeria Using *InvA* Gene Sequences

**DOI:** 10.3390/ani15233399

**Published:** 2025-11-25

**Authors:** Emmanuel O. Fadipe, Ludwig E. Hölzle

**Affiliations:** Department of Livestock Infectiology and Environmental Hygiene, Institute of Animal Sciences, Faculty of Agriculture, University of Hohenheim, 70599 Stuttgart, Germany

**Keywords:** *Salmonella enterica*, phylogenetic tree, genetic diversity, Genbank, Nigeria

## Abstract

*Salmonella* is a significant public health concern in Nigeria causing foodborne illnesses. Genetic diversity and prevalence of *Salmonella* is poorly understood in Nigeria. This study assessed the occurrence of *Salmonella* in various poultry houses in Nigeria and explored the evolutionary relationships among the isolates by analysis on *invA* gene. A total number of 314 samples (feces, feed, dust, waters, and palm swab) were collected from 49 farms in Abeokuta (18), Ibadan (20) and Oshogbo (11). *Salmonella* was detected with a prevalence of 2.75% in Ogun, 6.0% in Osun and 5.71%, in Oyo States, respectively. The overall prevalence of *Salmonella* in poultry farms sampled was 15/314 (4.78%). Sequence analysis revealed two sequences 01 and 02 to have 99.59% and 89.04 homologies with sequence of Paratyphi serovar (LC320032) and Enteritidis serovar (LC318423) in GenBank, respectively. Sequence 01 clustered with *S.* serovar Enteritidis from the USA, whereas Sequence 02 formed a distinct clade near *S.* serovar Typhimurium from Egypt. These findings underscore the public health significance of *S. enterica* in Nigeria, particularly in relation to food animals. The study highlights the need for improved farm management practices, stringent food safety regulations, and robust surveillance systems to mitigate the risk of *Salmonella* outbreaks.

## Simple Summary

*Salmonella* is a bacterium that can cause severe foodborne illness in humans and is often transmitted through contaminated poultry products. In this study, we collected samples from poultry farms in three states in southwestern Nigeria to determine the prevalence of *Salmonella* and to characterize the types of present. Out of 314 samples: including water, feed, feces, dust, and workers’ hands, 15 tested positive for *Salmonella*. We then analysed the genetic material of these isolates. Some were closely related to strains known to cause disease in other countries, while others appeared to be distinct but still potentially harmful. These findings are significant because they show that poultry farms in Nigeria can serve as reservoirs of *Salmonella* capable of transmission to humans through contaminated food. The study underscores the urgent need for improved hygiene practices, stronger food safety regulations, and routine monitoring on farms. Implementing these measures will help protect consumers, limit the spread of disease, and support safer poultry production in Nigeria.

## 1. Introduction

Poultry production is a major source of animal protein globally, including in Nigeria, where the poultry population increased from 151 million in 2005 to 192 million in 2010 [1,2]. Despite this growth, the sector faces persistent challenges such as disease outbreaks and biosecurity lapses. Among these, salmonellosis remains one of the most important zoonotic infections affecting poultry and humans [3,4,5]. *Salmonella enterica*, a Gram-negative bacterium of the family *Enterobacteriaceae*, is the predominant species responsible for both typhoidal and non-typhoidal infections in animals and humans [6,7].

In Nigeria, studies have reported varying prevalence of *Salmonella* across regions. For instance, Asogwa et al. [8] detected the pathogen in chickens in Enugu State, while Jibril et al. [9] identified *Salmonella* from environmental samples and farm workers’ shoes in Northwestern Nigeria. In contrast, few studies have explored *Salmonella* diversity in Southwestern Nigeria, with available data limited to isolates from post-mortem samples or cloacal swabs [10,11]. Consequently, surveillance data on the circulating strains and their genetic diversity in live poultry and farm environments remain scarce. Culture-based detection remains the gold standard for identifying *Salmonella*, but it is time-consuming and labor-intensive. Polymerase Chain Reaction (PCR) targeting the *invA* gene that is located on pathogenicity island 1 (SPI-1) offers a rapid and reliable diagnostic alternative due to its high specificity for *Salmonella* species [12,13,14,15,16]. Despite the importance of such molecular tools, their application in active *Salmonella* surveillance within poultry systems in Southwestern Nigeria remains limited. Hence, the *invA* gene was targeted in this study because it is a highly conserved component of Salmonella Pathogenicity Island-1 (SPI-1) and encodes a key protein of the type III secretion system involved in epithelial cell invasion. Owing to this high level of conservation across *Salmonella* serovars, *invA* has been widely adopted as a reliable species-specific marker for molecular identification [12,13]. Beyond its diagnostic value, previous studies have shown that *invA* contains informative polymorphic sites that can support the differentiation of closely related isolates and provide useful phylogenetic signal [17]. Although phylogenetic inference based on a single locus presents inherent limitations, the broad application, stability, and evolutionary relevance of the *invA* gene make it an appropriate marker for preliminary phylogenetic and molecular analyses of *Salmonella* in epidemiological studies.

Salmonella infections in poultry not only threaten animal health but also pose significant public health and economic challenges. High farm-level prevalence can lead to contaminated poultry products, contributing to foodborne illnesses and potential outbreaks in humans [18]. Risk factors influencing *Salmonella* occurrence include poor farm hygiene, inadequate biosecurity, presence of rodents, certain poultry breeds, and large-scale operations [1,10]. Additionally, the emergence of multidrug-resistant strains further complicates control efforts and threatens sustainable poultry production. Addressing these gaps requires robust surveillance systems, rapid detection methods, and better understanding of the circulating strains and their genetic diversity in local poultry populations.

Therefore, this study aimed to determine the prevalence of *Salmonella* in poultry farms across three Southwestern states and to characterize the genetic relatedness of isolates using *invA* gene sequencing. The findings from this research provide baseline data for improved monitoring, biosecurity, and public health risk assessment in Nigeria’s poultry production systems.

## 2. Materials and Methods

### 2.1. Study Area

**Figure 1:**
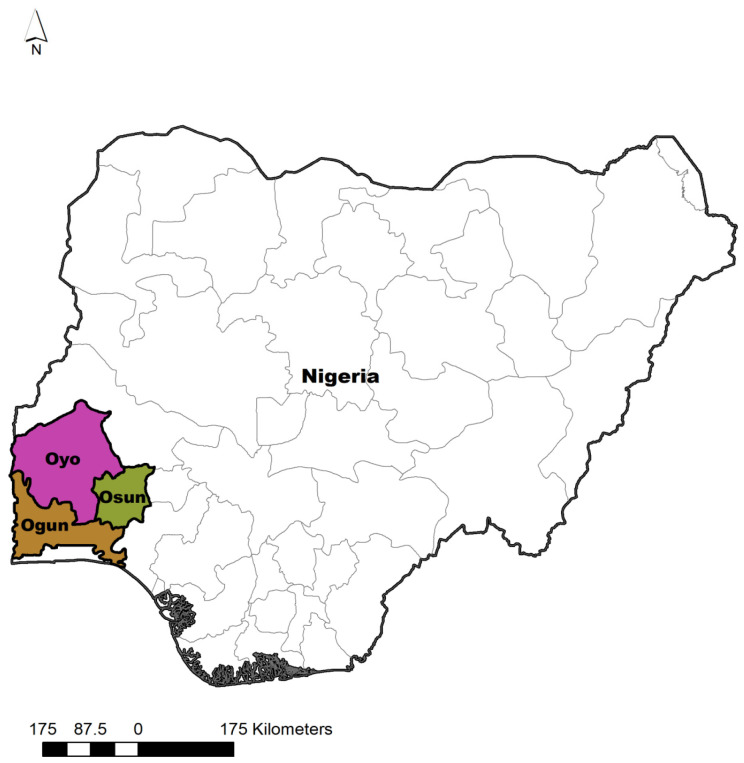
The map of Nigeria showing the States where the samples were collected.

This study was carried out in the southwestern zone of Nigeria. This zone includes Ogun, Oyo, Osun, Ondo, Ekiti, and Lagos. Except for Lagos, they are all endowed with both thick forest and derived savannah (Figure 1). A cross-sectional study employed a multistage sampling method in this survey. In the first stage, the Southwestern zone was purposely selected because it forms the central zone where large-scale poultry farming is practiced. In the second stage, three states (Ogun, Oyo, and Osun States) out of the six states in the Southwestern zone were selected by balloting without replacement. Second stage: All three chosen states have three senatorial districts. Hence, this resulted in the choice of one local government area from each senatorial district of the three states by random selection. In the third stage, one village was randomly selected as a sampling site from the local government chosen in each senatorial district. Hence, three villages were sampled per senatorial district, giving nine villages in each state. Random selection was ensured in those areas where the in-depth research could be achieved most effectively.

### 2.2. Sample Size Determination

Various studies have shown that the prevalence rate for salmonellosis in commercial poultry in Nigeria was 16%, which was used to determine the sample size. The sample size was calculated using the formula for cross-sectional studies [19]. The sample size for this study was selected from major areas within rural communities (i.e., at the grassroots level) where the primary livelihoods were based on agricultural and animal production practices.

### 2.3. Sample Collection

The samples collected from each farm included poultry feces, feed, dust, water, and palm swabs. A total of 314 samples were collected, which included 48 swabs from palms, 112 fecal samples, 51 water samples, 52 dust samples, and 51 feed samples. Sterile swabs were used to scrub the palms of the poultry workers, about 100 g per farm of dust was collected from different surfaces in each farm, commercially manufactured boot swabs (Technical Services Consultant, Lancashire, UK) were used to collect litter samples, fresh fecal dropping (about 100 g) collected from each farm, 100–200 g feeds were collected from different feeding troughs in the same farm and unmedicated water (100 mL) sample obtained from different poultry houses of the same farm. The samples were immediately transported in the mobile refrigerator (4 °C) to the Microbiology Laboratory of the College of Veterinary Medicine, Federal University of Agriculture, Abeokuta, for further analysis. The consent of the farmers was obtained through the Ethical Committee of the College of Veterinary Medicine at the Federal University of Agriculture, Abeokuta.

### 2.4. Isolation of Salmonella

All the samples collected were emulsified in sterile water. The culture and isolation of Salmonellae from the collected emulsified samples were carried out as described by Mshelbwala et al. [10]. Briefly, swabs, fecal, and environmental samples were pre-enriched in buffered peptone water and separately applied to the nutrient broth before incubation at 37 °C for 24 h. Two milliliters of the mixture were taken from the pre-enrichment medium and inoculated into 50 mL of Rappaport-Vasiliadis broth (Oxoid, Basingstoke, UK). The mixture was then transferred to tetrathionate glucose broth (Oxoid, Basingstoke, UK) for selective enrichment and incubated for 24 h at 37 °C. The typical colonies of *Salmonella* obtained from this culture were further sub-cultured on XLD agar and incubated for 24 h at 37 °C. The resulting *Salmonella*-suspected colonies were inoculated onto McConkey agar for purification. The cultures containing the growths or colonies were subjected to biochemical characterization for confirmation of *Salmonella*. The biotyping of the bacterial isolate was performed using standard biochemical characterization methods described by Cowan and Steel’s Manual for the identification of Medical Bacteria [20]. These included lactose oxidation, glucose fermentation, gas, hydrogen sulfide (H_2_S) production, the lack of β-galactosidase (ONPG), and lysine decarboxylation.

### 2.5. DNA Extraction from Salmonella Isolates

Three colonies from each of the positive cultures (subcultured three times to confirm clonality) were picked with a sterile loop and dissolved in 200 µL of distilled water to confirm the presence of Salmonella by biochemical characterization. DNA was extracted from the suspended culture using a Quick-DNA Fungal/Bacterial Miniprep Kit according to the manufacturer’s instructions (Zymo Research, Tustin, CA, USA). The eluted DNA was kept at 20 °C until use.

### 2.6. Amplification of the invA Gene of Salmonella

The invasive gene of *Salmonella* (*invA* gene) was targeted for amplification using a pair of primers described by Jibril et al. (2020) [9]; *invA* forward: GTGAAATTATCGCCACGTTCGGGCA and *invA* reverse: ATCGCACCGTCAAAGGAACC (synthesized by Bioneer Incorporation, Daejeon, Republic of Korea). The 25 μL final volume for the PCR reaction contained 12.5 μL of One Taq Quick-Load 2 × Master Mix (New England BIOLAB, Ipswich, MA, USA), 1 μL each of forward and reverse primers, 9.5 μL of nuclease-free water (New England BIOLAB), and 1 μL of template DNA. The reactions were placed in a Personal Cycler Series thermocycler (Biorad, Hercules, CA, USA). The reaction conditions were as follows: Initial denaturation at 94 °C for 4 min followed by 35 cycles of 94 °C for 1 min, 55 °C for 1 min, and 72 °C for 1 min; and final extension at 72 °C for 10 min. Ten microliters of the PCR products were subjected to electrophoresis through a 1% agarose gel in 1 × TAE buffer at 90 V for 60 min, along with 10 µL of GENEMate Quanti-Marker 100 bp DNA ladder (BioExpress, Kaysville, UT, USA). Gels were stained with ethidium bromide (Phenix Research Products, Candler, NC, USA) at a concentration of 5 µL/100 mL in the agarose gel suspension. After electrophoresis, the gel was visualized using a UV transilluminator and photographed with a handheld camera (Samsung, Huizhou, China). All positive samples were tested twice to confirm the PCR diagnosis. Positive DNA samples obtained from the pathogen laboratory of the Department of Veterinary Microbiology, College of Veterinary Medicine, Federal University of Agriculture, Abeokuta, and negative (nuclease-free water) samples were used as controls in each run.

### 2.7. DNA Sequencing of the invA Gene of Salmonella Isolates

To confirm and validate our results, fourteen culture-positive samples of *Salmonella* were selected, and their PCR products were sequenced directly using the Big Dye Terminator Cycle Sequencing Kit (Applied Biosystems, Foster City, CA, USA) with the forward amplification PCR primers and AmpliTaq-FS DNA Polymerase. The sequences obtained were viewed and compared using Finch TV and Sequence Scanner (Applied Biosystems) before being aligned with published sequences of various *Salmonella* species using Molecular Evolutionary Genetic Analysis software (MEGA 5.05).

### 2.8. Sequence Alignment and Analysis

The invA gene sequences of the isolated *Salmonella* were used to perform a BLAST search using the NCBI BLAST web server (https://www.ncbi.nlm.nih.gov/, accessed on August 2025). For comparison, the *invA* gene sequences of *Salmonella* isolates were obtained from the GenBank database using predefined selection criteria to provide appropriate phylogenetic context for the study isolates. Sequences were chosen to represent a broad geographical spread (Africa, Asia, North America), and to include isolates from different host sources such as poultry, human, and environmental samples. Also, a range of clinically and epidemiologically relevant serovar (including *S. enterica* serovars Enteritidis, Typhimurium, Gallinarum and related variants) was incorporated with preference given to enteries with available collection or submission dates to ensure inclusion of relatively recent isolates. The sequence accession numbers used to construct a phylogenetic tree are listed in Table 1. The alignment was done using the ClustalW method of Molecular Evolutionary Genetic Analysis (MEGA) software version 5.05 [21]. A phylogenetic tree was constructed using the Maximum likelihood method (ML) algorithm of the phylogeny program of MEGA 5.05 [21], which included two consensus sequences from this study and twelve sequences obtained from the GenBank with *Citrobacter freundii* (MZ202354) as the out-group to root the *invA* gene trees. The bootstrap confidence interval of the tree was determined based on 1000 replicates.

### 2.9. Statistical Analysis

The data were analyzed using descriptive statistic to determine the prevalence of *Salmonella* by sample type and location. Prevalence was expressed as percentage with 95% confidence interval calculated using the exact binomial method. Association between *Salmonella* occurrence and categorical variables were tested using the Chi-square test. A *p*-value < 0.05 was considered statistically significant.

## 3. Results

### 3.1. Prevalence of Salmonella in Samples Collected from Poultry Farms and Their Handlers

A total of 314 samples, including swabs from the palms of attendants, feces, water, and dust from 49 farms, were collected from Abeokuta (18), Ibadan (20), and Oshogbo (11). The samples were selectively cultured for 24 h to obtain typical *Salmonella* colonies. The overall prevalence of *Salmonella* in poultry farms sampled was 15/314 (4.78%), comprising 3 (6.25%) from 48 palm swabs from the poultry attendants, 4 (3.57%) from 112 fecal samples collected from the poultry houses, 5 (9.80%) from the 51 water samples, and 3 (5.88%) from the 51-feed sample collected (Table 2).

In all 52 dust samples collected around the poultry houses, *Salmonella* could not be detected. *Salmonella* species were found in the three sampled states, with prevalence rates of 2.75% (CI: 0.00–5.82), 6.0% (CI: 1.35–10.65), and 5.71% (CI: 1.27–10.15) in Ogun, Osun, and Oyo States, respectively. This revealed the highest prevalence in Osun State (Table 3).

### 3.2. Further Characterization of Salmonella Isolates by invA Gene Amplification and Sequencing

**Figure 2:**
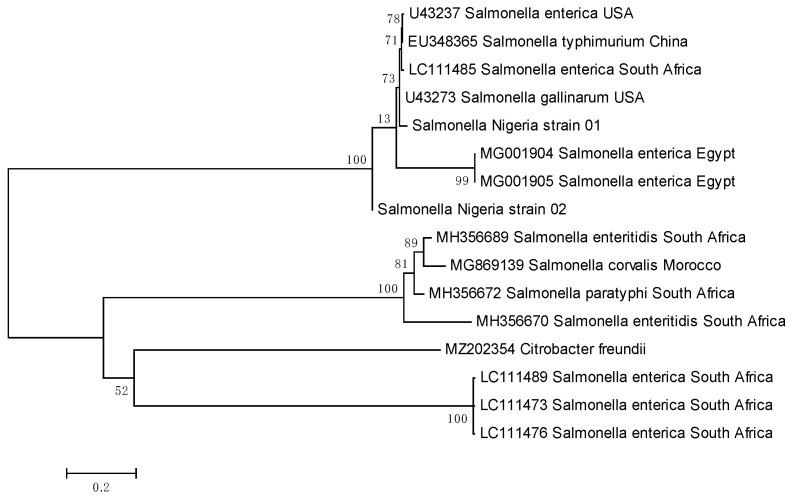
Evolutionary relationships of *salmonella* isolates found in this study compared to other sequences from the GenBank, using *invA* DNA sequences analyzed by the ML method. The percentage of replicate trees in which the associated taxa are clustered together in the bootstrap test (1000 replicates) are shown next to the branches in those. The tree is drawn to scale, with branch lengths in the same units as those of the evolutionary distances used to infer the phylogenetic tree. Source: Field Survey (2025).

DNA was extracted from the *Salmonella* colonies, amplified (*invA* gene), and the amplicons sequenced unidirectionally to characterize further and prove the genetic diversity of the isolates. Gel electrophoresis of the amplified PCR products derived from the DNA of the culture isolates reveals fourteen samples with visible bands of about 284 bp, the expected band size of *Salmonella enterica*. The PCR product of the negative control showed no band. Only two of the obtained sequences were usable and subjected to a BLAST search for homology in the GenBank, as the other sequences obtained were noisy. One revealed homology of about 99.59% with a sequence with accession number LC320032, a Paratyphi serovar, and the other revealed homology of about 89.04% with accession number LC318423, an *Enteritidis serovar* strain SYCH in GenBank. Analysis of the two sequences showed that sequence 01 has a 248 bp length and sequence 02 has 225 bp length with 51.4% and 51.6% mean G-C contents, respectively. Alignment of the two sequences with each other revealed that Sequence 01 has nucleotide G inserted at point 81, CCCG inserted at points 101–104, GGTA inserted at points 127–130, TGA inserted at points 155–157, TTAT inserted at points 167–170 and GT inserted at 229–230. The sequences of sample 01 and 02 have been deposited in GenBank with accession number PV879624 and PV879625, respectively. The phylogenetic tree inferred from the *invA* gene sequences of the *S. enterica* separated the isolates from this study into two clades, with isolate 01 tightly clustered the sequence of *S. enterica* serovar Enteritidis from the USA while isolate 02 is separated into a lonely clade but next to *S. enterica* serovar Typhimurium from Egypt (Figure 2).

## 4. Discussion

*Salmonella* is a critical pathogen in food animals in Nigeria due to its substantial impact on public health and the agricultural sector [22,23]. It is commonly found in livestock and poultry, where poor animal husbandry practices, such as inadequate sanitation and overcrowding, facilitate its spread. The contamination of meat, eggs, and dairy products with *Salmonella* poses a significant risk to human health, leading to foodborne illnesses that can range from mild gastroenteritis to severe systemic infections [24,25,26]. Additionally, the misuse of antibiotics in animal agriculture has led to the emergence of antibiotic-resistant strains, complicating treatment options and exacerbating the public health threat [27,28]. Addressing this issue requires comprehensive measures, including improved farm management practices, stringent food safety regulations, and robust surveillance systems to monitor and control the pathogen. Hence, to understand the prevalence and circulating *Salmonella* spp. in and around commercial poultry farms in Southwest Nigeria, this study assessed the possible sources of contaminants and characterized the detected Salmonella by sequencing and sequence analysis of the *invA* gene.

Furthermore, the isolation of *Salmonella* spp., which has been associated with zoonotic transmission of invasive non-typhoidal *Salmonella* (iNTS), is indeed worrisome. *S. Stanleyville* is mainly detected in poultry and cattle [1,29,30], but reports have associated this serovar with invasive human infections in West and Central Africa [31,32], and several outbreaks in Europe [33]. *S. virchow* seems associated with poultry [34,35] and accounted for 25% of NTS human infections in Australia [36]. In this study, a high prevalence of *Salmonella* infection (47.9%) was observed in commercial poultry farms in Nigeria. The results confirm observations from other parts of Nigeria by Fagbamila et al. (2017) [1], who showed 43.6% farm prevalence in commercial layer farms. Relatively high farm prevalence has also been reported in other sub-Saharan countries such as Ghana (44.0%), Uganda (20.7%), and Ethiopia (14.6%) [34,37,38] and likewise in developing Asian countries with reports of 46.3% and 18% prevalence in central Vietnam and Bangladesh, respectively [39,40].

The prevalence of *Salmonella* in Nigeria has been documented in several studies, showing varying rates. Recent investigations in southwestern Nigeria have also highlighted substantial Salmonella burdens in poultry farms, particularly in Osun and Ogun States [41]. For instance, a prevalence of 21.4% was reported in poultry cloacal swab samples [11] collected from Oyo State, prevalence of 81.1% in intestinal organs submitted for post-mortem from Lagos, Ogun, and Oyo States to the Veterinary Teaching Hospital [10], prevalence of 54% in fecal droppings of chicken from various farming systems [8] from Imo State, 15.9% prevalence in North West (Kebbi, Sokoto and Zamfara States) [9], 14% prevalence from eggshell and its content from Ogun State [42] and 8.6% from poultry intestinal content sampled in Ilorin, Kwara State [43]. These figures are higher than the 4.78% recorded in our study, indicating that the prevalence in Nigeria is relatively elevated. Several factors could be attributed to the results obtained from this study. Asogwa et al. (2022) [8] and Orum et al. (2022) [11] collected their samples, fecal droppings (120) and cloaca swabs (360), respectively, solely from a source that is believed to be richer in intestinal microbes, including *Salmonella* species. Biosecurity practices and farmers’ handling behaviour remain major determinants of Salmonella persistence in poultry environments, as recently demonstrated in Oyo State [44].

This contrasts with many developed countries, such as Poland, where the total percentage of infected flocks was 1.57%, and a decrease in the prevalence of *Salmonella* spp. in broiler chickens was observed, from 2.19% in 2014 to 1.22% in 2016. The reduction in European member countries can be attributed to the implementation of specific control programs [29], which are lacking in developing countries like Nigeria Large scale farms were found to have higher *Salmonella* sample prevalence compared to other categories of farm levels, indicating that once large farms were infected, the infection became more widespread in this farm type. Adesiyun et al. (2014) [45] observed a similar tendency for large farms compared to other farm categories from Caribbean countries. This might be attributed to the large number of the flock, making it difficult for the farmer to adhere to strict farm biosecurity and good farm management practices. The observation is not surprising, since there is conclusive evidence from the European Food Safety Authority that larger poultry farms have a higher chance of increased occurrence, persistence, and spread of *Salmonella* [46,47] than smaller farms. Furthermore, layer flocks, which spend a longer time in the poultry house, had a higher prevalence of *Salmonella* infection compared with broiler flocks.

In contrast, our study’s samples (314) were obtained from various sources, including dust, palm swabs, water, fecal droppings, and feed. This suggestion is further supported by the report of Ibrahim et al. (2020) [48] from Nasarawa State, which reported a higher prevalence of *Salmonella typhimurium* in diarrheic fecal samples compared to non-diarrheic samples. Additionally, the higher prevalence reported by others may be attributed to the larger number of samples collected and analyzed [49]. Another study that focused on the prevalence in commercial chicken eggs reported a rate of 6.5%, further highlighting the variability in prevalence depending on the specific sample and location within the region [49,50]. These variations underscore the importance of localized studies in accurately determining the prevalence of *Salmonella* and, consequently, guiding appropriate interventions.

Studies from other regions in African countries have shown even higher prevalence rates than our study. For instance, Edward et al. (2023) [50], Ramtahal et al. (2022) [51], and Waktole et al. (2024) [52] reported a prevalence of 32.1%, 14.4%, and 5.5% in South Africa, Egypt, and Ethiopia, respectively, except the study of Bouchrif et al. (2009) [53], which reported an extremely lower prevalence (0.91%) in Morocco. This study attempted to amplify and analyze the sequences of the *invA* gene that encodes a protein that is part of the Type III secretion system (T3SS), essential for injecting effector proteins into host cells, correlating to the virulence of Salmonella. The *invA* gene is highly conserved in *Salmonella* and is often targeted in PCR-based methods due to its specificity and reliability [12,54,55].

Molecular characterization of morphologically confirmed *Salmonella* isolates revealed a band size of 284 bp expected for the PCR product of the *invA* gene. This confirms the presence of *Salmonella* at the genus level in the study area. Our results agree with the findings of Naik et al. (2015) [56], Kaushik et al. (2014) [57], and Abhadionmhen et al. (2023) [58]. The *InvA* gene was amplified and sequenced in fourteen isolates in this study. While the inability to amplify the *InvA* gene in one of the samples may be associated with poor DNA quality, presence of inhibitors or primer mismatches, it may also suggest that the strain from which the *InvA* gene was not amplified may have undergone mutation [59] and not been invasive in nature; however, Naik et al. (2015) [56] and Kadry et al. (2019) [17] postulated that it may have another invasive mode of infecting its host. Though many studies suggest that all *Salmonella* species have the *InvA* gene [54,55,60], various studies in Nigeria could not achieve 100% amplification of this gene in all the *Salmonella* species analyzed in their studies, suggesting those not amplified were mutants. Still, the studies of Mokgophi et al. (2024) [61] in South Africa and Shi et al. (2012) [62] in China reported 100% amplification of the same gene in their studies. In the reports of Abhadionmhen et al. (2023) [58], Igbinosa et al. (2022) [23], and Raufu et al. (2021) [63] on amplification of *InvA* gene carried out in various States in Nigeria and Kadry et al. (2019) [17] in Egypt, *InvA* gene was not amplified in all the Salmonella species subjected to PCR amplification. Hence, if the gene is indeed not present or has mutated in some of the *Salmonella* species, then it can be suggested that the *InvA* gene is not a good candidate for routine diagnosis of salmonellosis and their genetic diversity. Various literature that supports [61] or does not support [64] the use of the *InvA* gene as a good candidate for Salmonella diagnosis and molecular characterization exist. Therefore, our study’s finding may also support the suggestion that the *InvA* gene may not be a good target for diagnosing salmonellosis in Nigeria, as many reports from the country revealed that not all the Salmonella isolates were amplified targeting the *InvA* gene amplification.

The phylogenetic analysis of the two *Salmonella* sequences obtained in this study, compared with reference sequences from GenBank, revealed their separation into two distinct clades, indicating genetic divergence between the isolates. This observation aligns with BLAST search results, which showed high sequence homologies (99.59% and 89.04%) with *Salmonella enterica* serovars *Paratyphi* and *Enteritidis*, respectively. Although only a limited number of sequences were successfully analyzed, these findings suggest the possible circulation of diverse *Salmonella enterica* strains within poultry environments in Southwestern Nigeria. *Salmonella enterica* serovar *Enteritidis* is a well-documented foodborne pathogen associated with gastroenteritis in humans, characterized by symptoms such as diarrhea, fever, abdominal pain, and vomiting [22,24,65]. Similarly, *S. enterica* serovar *Paratyphi* is implicated in paratyphoid fever, a systemic illness resembling typhoid fever [66]. While the present data are limited, the detection of these serovars points to potential public health implications and emphasizes the need for improved farm hygiene and biosecurity. Globally, *S. Enteritidis*, *S. Typhimurium*, and *S. Heidelberg* have been among the most frequently reported serotypes causing human salmonellosis in North America and Europe [67,68]. The occurrence of related strains in this study area reinforces the importance of continuous surveillance and molecular monitoring to better understand *Salmonella* diversity and its implications for poultry and public health.

## 5. Conclusions

The detection of *Salmonella enterica* in poultry environments across Southwestern Nigeria indicates potential public health risks within poultry production systems. However, given the limited number of isolates, partial *invA* sequences, and absence of serotyping or antimicrobial susceptibility testing, these findings should be interpreted cautiously. They provide preliminary evidence rather than definitive conclusions about serovar distribution or epidemiological patterns. Future studies should include expanded and longitudinal sampling, detailed serotyping, and antimicrobial susceptibility testing following CLSI or EUCAST guidelines. Incorporation of additional molecular targets or whole-genome sequencing, along with risk factor analysis using appropriate regression models, will enhance understanding of *Salmonella* ecology in poultry systems. Also, strengthening biosecurity education, routine surveillance, and collaborative monitoring among farmers, veterinarians, and regulatory agencies remains essential to mitigate potential zoonotic transmission and improve food safety.

Recommendations from the Research:

1. Enhanced Surveillance: Implement systematic monitoring of *Salmonella* in poultry farms across Southwestern Nigeria. Molecular detection using *invA* gene PCR should be integrated into routine diagnostics to ensure early and accurate identification.
2. Farm Biosecurity Measures: Poultry farmers should adopt strict biosecurity protocols, including controlled access, regular cleaning and disinfection, safe feed and water handling, and minimizing contact between domestic and wild birds.
3. Farmer Education and Training: Conduct regular training to educate poultry farmers on *Salmonella* risks, prevention strategies, hygiene practices, and vaccination benefits.
4. Food Safety Measures: Strengthen regulations for proper handling, processing, and cooking of poultry products to reduce human infection risk. Food safety inspections and public compliance should be emphasized.
5. Policy and Regulatory Support: Government and public health authorities should enforce policies for disease surveillance, outbreak response, farm inspections, and zoonotic disease control.
6. Future Molecular Studies: Encourage further molecular epidemiological research to:
  - Characterize the genetic diversity of *Salmonella* strains in poultry and humans.
  - Detect virulence and antimicrobial resistance genes beyond *invA*, such as *stn*, *hilA*, and *spiC*.
  - Use whole-genome sequencing (WGS) and multilocus sequence typing (MLST) to track transmission pathways, understand evolution, and inform vaccine development.
7. Public Health Awareness: Promote campaigns linking poultry health to human health to prevent zoonotic transmission.
8. Stakeholder Collaboration: Strengthen cooperation among veterinarians, public health professionals, researchers, and farmers for integrated disease control strategies.

## Figures and Tables

**Table 1:** Salmonella reference sequences of *invA* gene and accession numbers obtained from the GenBank.

No	Species	Subspecies	Serovar	Strain	Accession No.
1	*S. enterica*	*enterica*	Typhi	R19.2839	CP046429
2	*S. enterica*	*enterica*	Gallinarum	RKS2962	U43273
3	*S. enterica*	*enterica*	Haifa	EGY 2	MG001905
4	*S. enterica*	*enterica*	Typhimurium	EGY 1	MG001904
5	*S. enterica*	*enterica*	Typhimurium	SALS 7	LC111485
6	*S. enterica*	*enterica*	Typhimurium	CVCC541	EU348365
7	*S. enterica*	*enterica*	Typhimurium	RKS4194	U43237
8	*S. enterica*	*enterica*	Corvallis	25B	MG869139
9	*S. enterica*	*enterica*	Paratyphi	JQ694526	MH356672
10	*S. enterica*	*enterica*	Enteritidis	CP018657	MH356689
11	*S. enterica*	*enterica*	Enteritidis	CP018655	MH356670
12	*S. enterica*	*enterica*	Enteritidis	NCCP16206	CP041973

Source: Field Survey (2025).

**Table 2:** Variation in prevalence of *Salmonella* based on sample types collected from poultry farms in Abeokuta, Oshogbo, and Ibadan, Nigeria.

No	Sample	No Collected (%)	No Positive (%)	95% CI
1	Swabs	48	03 (6.25)	0.00–13.1
2	Feces	112	04 (3.57)	0.13–7.01
3	Water	51	05 (9.80)	1.64–17.97
4	Dust	52	00 (0.00)	0.00–0.00
5	Feed	51	03 (5.88)	0.00–12.34
6	Total	314	15	2.42–7.14

Source: Field Survey (2025).

**Table 3:** Prevalence of Salmonella species based on the location of the samples collected.

Location	No of Farm Sampled	Total No of Sample	No Positive (%)
Abeokuta	18	109	03 (2.75%)
Oshogbo	11	100	06 (6.00%)
Ibadan	20	105	06 (5.71%)
Total	49	314	15 (4.78%)

Source: Field Survey (2025).

## Data Availability

GenBank PV879624-PV879625.
